# A Framework for Applying Natural Language Processing in Digital Health Interventions

**DOI:** 10.2196/13855

**Published:** 2020-02-19

**Authors:** Burkhardt Funk, Shiri Sadeh-Sharvit, Ellen E Fitzsimmons-Craft, Mickey Todd Trockel, Grace E Monterubio, Neha J Goel, Katherine N Balantekin, Dawn M Eichen, Rachael E Flatt, Marie-Laure Firebaugh, Corinna Jacobi, Andrea K Graham, Mark Hoogendoorn, Denise E Wilfley, C Barr Taylor

**Affiliations:** 1 Leuphana University Institute of Information Systems Lueneburg Germany; 2 Palo Alto University Center for m2Health Palo Alto, CA United States; 3 Stanford University Department of Psychiatry and Behavioral Sciences Stanford, CA United States; 4 Washington University in St Louis Department of Psychiatry St Louis, MO United States; 5 University at Buffalo Department of Exercise and Nutrition Sciences Buffalo, NY United States; 6 University of California San Diego Department of Pediatrics San Diego, CA United States; 7 University of North Carolina at Chapel Hill Department of Psychology and Neurosciences Chapel Hill, NC United States; 8 Technische Universität Institute of Clinical Psychology and Psychotherapy Dresden Germany; 9 Northwestern University Department of Medical Social Sciences Chicago, IL United States; 10 Vrije Universiteit Department of Computer Science Amsterdam Netherlands

**Keywords:** Digital Health Interventions Text Analytics (DHITA), digital health interventions, eating disorders, guided self-help, natural language processing, text mining

## Abstract

**Background:**

Digital health interventions (DHIs) are poised to reduce target symptoms in a scalable, affordable, and empirically supported way. DHIs that involve coaching or clinical support often collect text data from 2 sources: (1) open correspondence between users and the trained practitioners supporting them through a messaging system and (2) text data recorded during the intervention by users, such as diary entries. Natural language processing (NLP) offers methods for analyzing text, augmenting the understanding of intervention effects, and informing therapeutic decision making.

**Objective:**

This study aimed to present a technical framework that supports the automated analysis of both types of text data often present in DHIs. This framework generates text features and helps to build statistical models to predict target variables, including user engagement, symptom change, and therapeutic outcomes.

**Methods:**

We first discussed various NLP techniques and demonstrated how they are implemented in the presented framework. We then applied the framework in a case study of the Healthy Body Image Program, a Web-based intervention trial for eating disorders (EDs). A total of 372 participants who screened positive for an ED received a DHI aimed at reducing ED psychopathology (including binge eating and purging behaviors) and improving body image. These users generated 37,228 intervention text snippets and exchanged 4285 user-coach messages, which were analyzed using the proposed model.

**Results:**

We applied the framework to predict binge eating behavior, resulting in an area under the curve between 0.57 (when applied to new users) and 0.72 (when applied to new symptom reports of known users). In addition, initial evidence indicated that specific text features predicted the therapeutic outcome of reducing ED symptoms.

**Conclusions:**

The case study demonstrates the usefulness of a structured approach to text data analytics. NLP techniques improve the prediction of symptom changes in DHIs. We present a technical framework that can be easily applied in other clinical trials and clinical presentations and encourage other groups to apply the framework in similar contexts.

## Introduction

Digitally delivered interventions for mental disorders have the potential to reduce the mental health burden worldwide [1]. Efficacious online and mobile phone app–based programs can overcome barriers to treatment such as stigma, reach, access, cost, and the scarcity of professionals trained in empirically supported interventions [2]. Furthermore, digital health interventions (DHI) are more scalable, potentially allowing one professional to manage a large number of individuals [3]. As DHIs are increasingly used, new data analytics capabilities are needed to evaluate treatment outcomes and mechanisms of engagement and symptom reduction [4].

Most DHIs collect structured data that are pertinent to assessing adherence to the intervention and symptom change over time, including symptom severity scales, number of sessions completed, and number of times the program was accessed [5]. Digital guided self-help interventions, a type of DHI, also incorporate a trained practitioner (*coach*) who facilitates the user’s learning of the intervention material, monitors progress, and helps troubleshoot barriers to change. This allows for the collection of rich, in-depth text data that could augment the understanding of intervention efficacy and inform the development and refinement of future programs. Such datasets include texts generated through direct communication between users and their facilitators through a digital platform. Another source of information comes from text users’ record during the intervention, for example, free-text diary entries and posts authored on intervention-related group chats and discussion boards [6]. Data analytic approaches, therefore, could benefit from cultivating an overarching perspective on methods to apply for studying the text data emerging from technology-delivered programs.

Hereafter, we provide a brief review of the use of text analytics methods in DHIs. Then, we propose a framework for applying natural language processing (NLP) in this field and demonstrate its application in a test case of an online intervention for eating disorders (EDs), delivered as part of the Healthy Body Image (HBI) Program trial [7].

## Methods

### Natural Language Processing in Mental Health Interventions

NLP is a rapidly evolving interdisciplinary field that studies human language content and its use in predicting human behavior [8]. NLP models utilize computational models to analyze unstructured, user-generated text to identify patterns and related outcomes (eg, a change in target symptoms) [9]. If proven effective, NLP models may ultimately enable the design of automated chatbots in person-machine communication [10]. Although the use of NLP in consumer and online search behavior is well established [11], it has only recently been utilized in mental health research [12].

Text data analytics can inform clinical decisions, particularly when professionals have many data points at their disposal, but each characteristic has weak predictive potency [13]. Using NLP models, researchers have evidenced, for instance, that text communications can predict an increase in psychiatric symptoms [14], that text data on electronic medical records can effectively predict treatment outcomes [5], and that patients’ reviews of the care they receive can provide important insights for stakeholders [15]. Furthermore, when analyzing text data, machine learning algorithms demonstrated greater accuracy than mental health professionals in distinguishing between suicide notes written by suicide completers and controls [16]. A similar approach has also been utilized in understanding medical risks through NLP of electronic medical records [17].

NLP strategies have also been applied to analyze text data from social media in the context of mental health. For instance, Coppersmith et al [18] detected quantifiable signals of mental disorders through analyses of text data available on Twitter. NLP is also effective in using text messages exchanged with a crisis intervention service to predict outcomes [8]. Computational discourse analysis methods have been employed to develop insights on what constitutes effective counseling text conversations as well [19]. Similarly, by analyzing patterns of the words, sentiments, topics, and style of messages used, Hoogendoorn et al [12] found a correlation between several text features and social anxiety in an online treatment. However, research on the clinical applicability of NLP models is still in its early stages [10]. For example, Miner et al [20] have shown that currently available smartphone-based conversational agents (eg, Apple’s Siri), which many individuals use to search health information [21], are not equipped to respond effectively to users’ inquiries about mental health. Considering the potential of text data to inform and enrich both clinicians and clients, the development and refinement of NLP tools should be a significant public health priority.

### Proposed Framework

NLP offers a useful set of tools for analyzing text data generated in DHIs and for building predictive models. NLP can clarify the mechanisms mediating the effects of online interventions as well as improve and personalize DHIs, leading ultimately to further automation of technology-delivered programs and lower costs [22]. DHI’s free text may be created by 2 sources. First, information about users’ thoughts, emotions, and behaviors is collected via open-ended questions embedded within the program (eg, “Hey [user], after learning about triggers, can you identify two of your common triggers for binge eating?”). Employing NLP techniques to this type of text data can be used to build predictive models, for instance, for calculating individual mood symptoms and symptom trajectories [23]. Second, in guided self-help interventions, users and coaches exchange messages for problem solving, engaging users, providing supplemental information, and individualizing the intervention.

**Figure 1:**
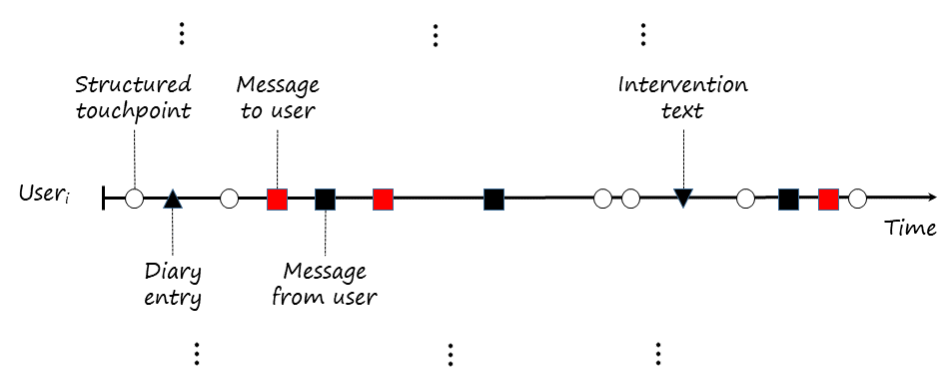
Text fragments along an exemplified user journey of a specific user i (vertical dots refer to other users); open circles refer to other nontext touchpoints and the interaction of the user with the digital health intervention; upward pointing triangles refer to fragments from diaries; red squares refer to the messages sent by coaches; black squares refer to the messages sent by users; and downward pointing triangles refer to the data collected within specific exercises (eg, deep breathing).

In DHIs, each text snippet, that is, a free-text segment, is associated with a specific user and has a unique time stamp. Figure 1 represents an exemplified *user journey* and shows the time interval a user spends within a DHI. Each filled symbol on the timeline represents a text snippet where the shape and color reflect the text classes (eg, a message from a user). Text snippets are not the only elements of user’s journeys; instead, structured touchpoints (indicated by open circles in Figure 1) complete the data associated with specific users. A touchpoint is, broadly speaking, an interaction of the user with the DHI. Besides text messages exchanged between users and coaches, this includes symptom severity scales.

**Figure 2:**
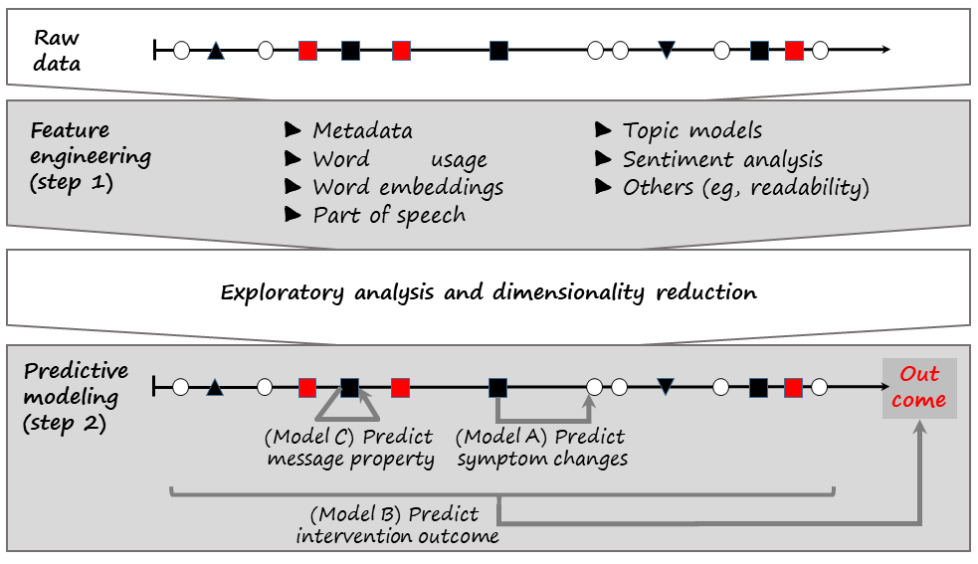
Framework for the analysis of textual data in DHIs (symbols are explained in the caption of fig. 1).

The analysis of texts in DHIs encompasses 2 steps (Figure 2). The first step, feature engineering, concentrates on preprocessing the text data to identify structured features (free texts cannot be directly used by machine learning algorithms). These features form a numerical vector of typically fixed length that represents each snippet and can be used to estimate statistical models. In the second step, predictive modeling, models are constructed to infer and predict either short-term symptom change or overall therapeutic outcomes. Information acquired in this step increases our understanding of the factors precipitating and maintaining primary mental health outcomes. These data also promote the refinement of DHIs, including automating key intervention components, such as in-program coaching or sending reminders to log in or self-record data.

### Step 1: Feature Engineering

The feature engineering focuses on preprocessing the text snippets (originating either from the intervention or the messages exchanged between the users and coaches). As the lengths of the intervention snippets and messages are likely to vary, we aimed to derive a fixed length vector that represents each text snippet in a structured way, that is, technically transforming all text snippets into either numbers or factors. In the following paragraphs, we describe the different classes of features that we implemented.

#### Metadata

Metadata features include descriptive qualities of text snippets that are content-agnostic and do not involve semantics [24]. Metadata encompass text-specific features such as the number and length of words, sentences and paragraphs, use of punctuation and special characters, the ratio of capital letters, and text layout (eg, indentation). Other metadata include the time stamp of when the text was authored and even its location. Metadata also include whether the text was composed as part of the intervention or sent spontaneously between the users and coaches.

#### Word Usage

Word usage indicates the use of specific terms. Preprocessing involves multiple actions such as tokenization (ie, splitting text into single terms), stemming/lemmatization (ie, mapping related terms to a common base form), converting terms to lower case, removal of frequently occurring terms (also known as *stop words*), and synonym substitution (refer to the study by Manning et al [25] for an excellent overview). Then, documented frequencies per word are determined, allowing for the removal of text snippets with very high or very low frequencies from the analysis, which might not be highly informative. With the remaining words, each text snippet is represented by a vector that contains the word’s specific counts. An aggregating feature is vocabulary richness (ie, how many different words are used). To extend this approach, the frequency of n-grams, that is, a sequence of words of length *n*, can be analyzed (for review of frequent pattern mining in texts, refer to the study by Zhong et al [26]).

#### Word Embeddings

Word embeddings represent (unique) words by low-dimensional numerical vectors [27]. This numerical representation is generated by analyzing large text corpora and studying the co-occurrences of words in documents. The hypothesis behind it is that words that co-occur in documents share some common characteristics. Pretrained word embeddings are available for many languages, utilizing recent computational advances to complete this task efficiently, for example, Word2Vec [28] and GloVe [29]. If each word of a text snippet is represented by an *n* dimensional vector, the snippet itself can be represented by a vector of this size by averaging elementwise over the *n* dimensions [30].

#### Part-of-Speech Tagging

Part-of-speech (POS) tagging assigns each word in a text snippet a class of word types (eg, noun, verb, and adjective) that not only depends on the word itself but also on its context. Current approaches and software packages [31] yield accuracies of POS classification greater than 95%. For generating POS features, we used the Apache OpenNLP library that categorizes words according to the Penn Treebank tag set [32]. Although in this paper we only employ POS tagging, named entity recognition [33] can also facilitate the identification of words that refer to persons or locations.

#### Topic Models

Topic models try to uncover a latent semantic structure of a collection of documents. For this purpose, we assume that each document in the collection is generated from several topics. Each topic can be characterized by a set of words. Latent Dirichlet Allocation (LDA) [34] is one of the prominent approaches to derive topics from a collection of documents. We apply LDA to the collection of all text snippets and assume that they were generated by N topics. Each text snippet can then be represented by an N-vector that illustrates the mixture of the topics identified by the LDA. Topic modeling is an active research field with many advances, one being guided LDA, which enables domain experts to define seed words for topics.

For *sentiment analysis* [33], dictionaries are used to identify words with positive or negative sentiment. In addition, some dictionaries, for example, the sentiment lexicon of the Research Council of Canada [35], enable the association of more granular emotions and single words (eg, joy, fear, and disgust). When using different dictionaries during the sentiment analysis, counting the number of positive and negative words (and other types of sentiments) in each text snippet adds new features for each of the dictionaries used. The number of new features reflects the number of sentiment types in the dictionaries used for this purpose.

There are other sources of features which we do not employ in the proposed analysis, given that they are likely less relevant for understanding outcomes in DHIs. For example, *readability* tries to measure how understandable and interesting a document is. There are also readability approaches that study the cohesion between sentences [36]. *Lexical diversity* also enriches the understanding of text snippets, and many corresponding metrics and software libraries have been developed, for example, the R package koRpus [37]. Finally, *spell checking* serves as a source to generate features, for example, the ratio of misspelled words (see software libraries such as Hunspell for details [38]).

Features derived from the *coach-user communication* offer additional information, for example, response times and frequencies [12]. Carefully measuring these features (and their dynamics) would require interpreting messages and categorizing them as questions and answers. Instead, we analyzed the sequence of coach/user messages without taking the message content into account and, then, counted how often a coach message is directly followed by a user message. For example, the sequence of coach-user communication might be CCUCUCUCCCUU (C=coach and U=user); here, 7 and 5 messages were sent by the coach and the user, respectively. Only 4 messages from the coach were followed directly followed by a user message, indicating a response rate of 4/7. In addition, we calculate the average time taken by a user to *respond* to her coach.

At the end of the feature engineering step, each text snippet is represented with numerous features derived from the above analyses. To make features comparable, those derived from word usage, word embeddings, POS tagging, and sentiment analysis are normalized by dividing them by the overall word count of each snippet. As a rule of thumb, if only little text data are available (ie, 5 times the number of features is greater than the number of text snippets), generic methods for dimensionality reduction should be applied, for example, principal component analysis.

### Step 2: Predictive and Inference Modeling

In step 2 (Figure 2), supervised learning approaches [39] are utilized to (A) infer symptom severity over time; (B) predict a therapeutic outcome, which could include premature dropout; and (C) infer message characteristics. These models are explained below:

- Model A—inferring symptom severity over time: Model A tries to establish an association between the symptom level and (temporally) adjacent text snippets. As the symptom measurements and text snippets form a sequence (as illustrated in Figure 1), one approach is to infer the symptom measurement from the text snippet that is closest in time (either before or after the text snippet was authored). An alternative route is to define a fixed length time window around a given text snippet and calculate the average over symptom scales in this time window.
- Model B—predicting a therapeutic outcome: Model type B focuses on predicting 1 target variable per user. For instance, one might want to know halfway through the intervention whether a user is likely to further improve, and what might help them do so. As these variables include only one outcome per user (ie, symptom level at the end of the intervention), the features generated on the level of single text snippets must be aggregated, including average, variance, and linear or nonlinear trends, over the course of the intervention for individual users. Such a trend metric could, for instance, represent how the average sentiment score per user evolves over time, which might ultimately be a predictor of the therapeutic outcome or the course of symptoms over time (model type A).
- Model C—inferring message characteristics: Text snippets can be associated with a set of characteristics. For instance, a user message might be either a question, a statement, or an answer to a previous question from the coach. Or, for example, we might have a scale for each text snippet that reflects the suicidal risk for a user. Models of type C take the text features of each snippet and try to infer whatever characteristic is of interest (this model type is not covered in the following case study and is mentioned here for completeness). As the text snippets are linked to individual users, hierarchical modeling approaches could be employed for model types A and C.

When predicting the therapeutic outcome, the number of features can be greater than the number of observations, that is, the number of users. To handle this situation, there are various approaches to select important features, from dedicated methods such as the least absolute shrinkage and selection operator (LASSO) regression (or the Bayesian analogue) to simple approaches such as backward and forward selection or methods that incorporate feature selection (eg, pruning of decision trees by cross-validation). In all analyses, a proper cross-validation of the models is key. Only looking at the correlations might overestimate the predictive power of specific features.

The statistical models derived can finally be utilized to inform therapeutic decisions [39], such as selecting the most effective intervention or the appropriate level of guidance. As these models do not necessarily reflect causal relationships and may be a product of endogeneity, they should be handled with care and might only serve as a basis to explore causality in subsequent randomized controlled trials (RCTs).

We implemented the above process as an R package called Digital Health Interventions Text Analytics (DHITA). The R code is available upon request from the authors. In the following section, we apply the above framework to the text data generated in a large-scale intervention study that focused on EDs.

## Results

### The Intervention

Student Bodies–Eating Disorders (SBED) was a digital guided self-help program for individuals with EDs, designed to reduce ED psychopathology and negative body image in college-age female students. The intervention comprised 40 core sessions that were self-paced and delivered online or via a specialized app over the course of 8 months. This guided self-help psychoeducational and cognitive behavioral therapy–based material was supplemented by the support of online mental health coaches who were graduate students in clinical programs, postdoctoral fellows, or study staff members under the supervision of licensed clinical psychologists. Coaches and their assigned users communicated via text messages, delivered through the SBED platform. Users were encouraged to contact their coaches with any questions, difficulties, dilemmas, and other issues relevant for their progress in the program. Coaches both responded to the messages they received from their assigned users and initiated text correspondence regarding the users’ progress in the program and the data that users recorded about their ED and related difficulties.

### The Studies

In this paper, we utilize data from 2 studies testing the SBED intervention. The HBI Program study is a large, multisite RCT testing the efficacy of SBED for college women with EDs. Students in 28 US universities and colleges who screened positive for an ED (other than anorexia nervosa, who received a medical referral) were randomized, at the school level, to either receive the intervention or a referral to care as usual at their respective college counseling/health center [40]. In addition, SBED was offered to college students in Missouri, United States, as part of a statewide implementation of the online platform used for screening and intervention in EDs [41]. In total, 372 college students participated in SBED across these initiatives and were assigned a coach with whom they could correspond. Overall, users in the combined dataset of both initiatives generated 37,228 intervention text snippets and sent 4285 messages to their coaches.

The DHITA framework could provide useful insights to clinicians and organizations implementing DHIs with their clients. For instance, data collected in model A could help flag a user who is more likely to relapse in the near future, thereby activating a set of targeted microinterventions and informing a case manager. As model A capitalizes on the data gathered implicitly (eg, by using adjacent text snippets), it can reduce the user burden. Similarly, the potential benefit of model B is that it can inform clinicians and stakeholders of the long-term outcomes and early dropout, for instance, by offering only these users a higher level of care. To increase the scalability of DHIs, some of the guidance provided in these programs should be automated; using machine learning techniques, model C could help researchers and developers distinguish between messages to which response could be fully or partly automated (eg, resolving technical inquiries) and messages that require a more nuanced and personalized response (eg, user reengaging after a break or needing immediate support).

### Feature Engineering (Step 1)

**Figure 3:**
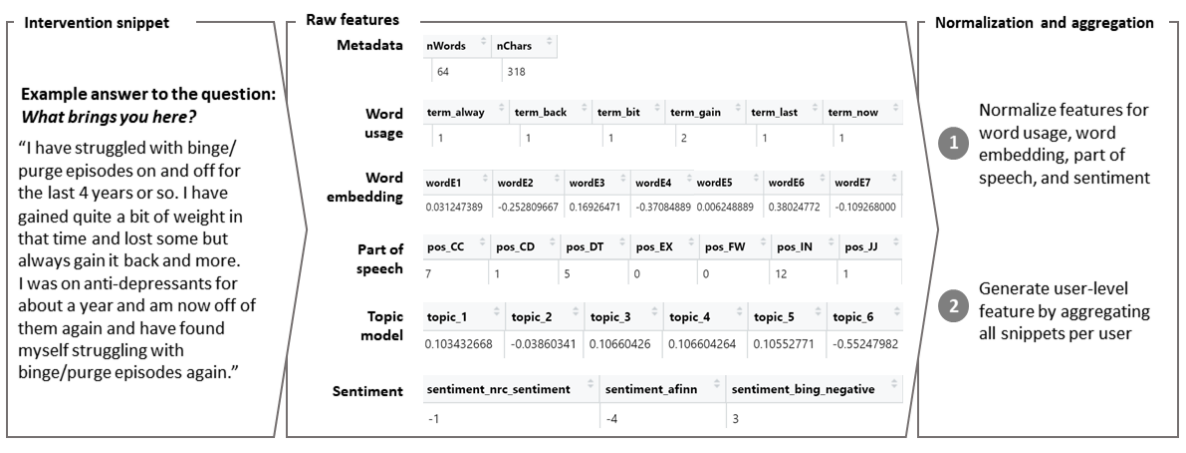
The figure presents an example for an intervention snippet. Raw features are derived as demonstrated by some selected features in each category (features describing the user-coach communication are not shown, because they are only defined on communication threads, but not individual snippets).

We applied the feature engineering to the 2 types of text data (intervention snippets and user messages) separately as they vary significantly in content and average length. An example is presented in Figure 3. As shown in Table 1, different hyperparameter choices, for example, the frequency thresholds for the proportion of word usage in all snippets to be included, impact the number of features derived, such as the representational dimension of the word embeddings. As a rule of thumb, in choosing hyperparameters for models A and C, we suggest maintaining more text snippets than features. Our choices in this study resulted in 200 and 310 features on the text snippet level for messages and intervention texts, respectively.

**Table 1:** Derived features to represent text snippets (we provide the full set of features to interested readers upon request).

Feature type	Number of features^a^	Comment	Examples (for message snippets)
Metadata	2|2	Number of words and characters	—^b^
Word usage	79|189	For messages: MINOCC^c^=0.05 and MAXOCC^d^=0.5; for intervention snippets: MINOCC=0.005 and MAXOCC=0.5	Most common words in approximately one-fourth of all messages: think, feel, eat, just, and like
Word embeddings	50|50	We used the pretrained GloVe with 50 dimensions and an average over each dimension as suggested by De Boom et al [30]	—
POS^e^	44|44	Note that for the intervention snippets it took approximately 10 hours to generate the POS features on 1 core of an Intel i7	Most common POS tags: personal pronouns, nouns, prepositions, particles, and determiners
Topic models	10|10	Probabilities for 8 topics+SD of these numbers+log likelihood	—
Sentiments	15|15	We used 3 different lexica: National Research Council Canada (NRC) (11), AFINN^f^ (1), and Bing^g^ (3), where numbers in parenthesis indicate the number of dimensions	NRC sentiment types: anticipation, trust, joy, sadness, and fear
Communication	2|0	Only available for message snippets (response rate and mean response time) and only aggregated on the user level	—

^a^The first number in this column refers to the number of features for the message snippets and the second refers to the intervention snippets.

^b^Not applicable.

^c^A specific term occurs in at least MINOCC of all messages (minimum occurrence).

^d^A specific term occurs in not more than MAXOCC of all messages (minimum occurrence).

^e^POS: part-of-speech.

^f^AFINN is an English word list developed by Finn Årup Nielsen. Words scores range from minus five (negative) to plus five (positive).

^g^Another list of words from the search engine Bing.

**Figure 4:**
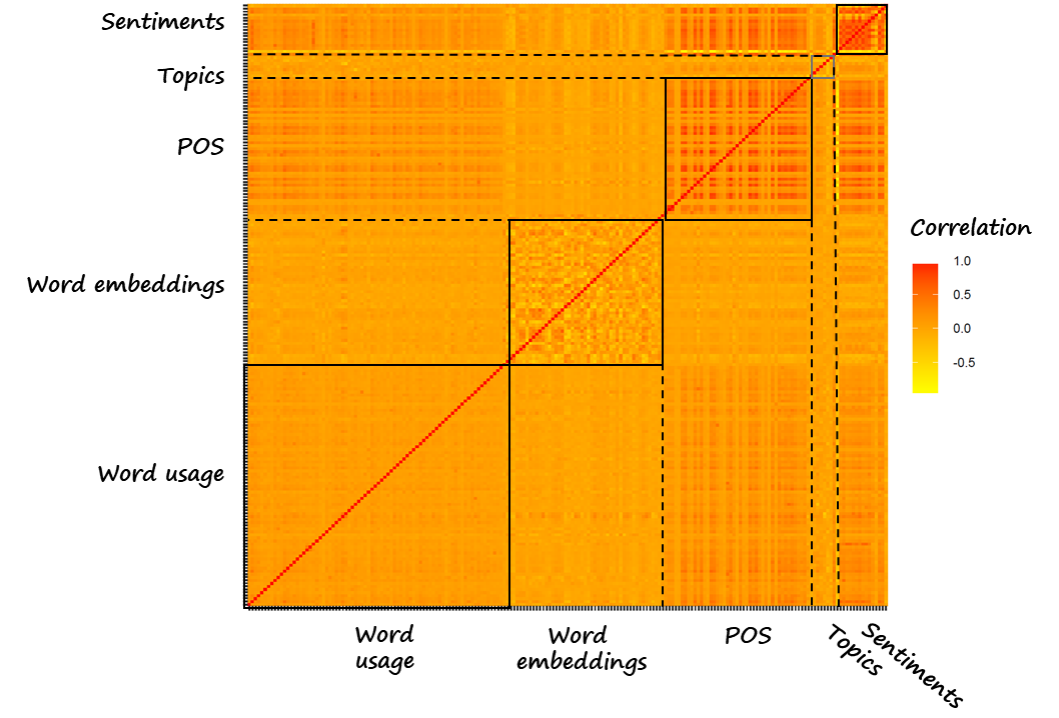
Correlation between the 200 features for all user messages. The blue lines indicate the different feature types. The red dots on the diagonal refer to the correlation of each feature with itself, ie, correlation = 1.

In our case study, each user message is represented by a 200-dimensional feature vector. Figure 4 presents the correlation among these features. In summary, the orange color indicates a low correlation among most features, suggesting that they might be independently valuable in predictive modeling of future symptoms. Of note, the correlation within some feature types tends to be higher, for example, sentiment features show a strong correlation with itself as we would expect.

Note that this set of features exists on the level of each text snippet, be it a message or an intervention snippet. It could be used for model type A or to predict outcomes or dropout on a user level (model B, Figure 2). For the latter scenario, features need to be aggregated on a user level. For this purpose, 2 aggregation functions were used: the mean (for all features), and for the sentiment features, the SD was included as well. Including the mean and the SD may help to examine a potential future hypothesis about whether greater variability predicts less improvement over time.

### Predictive and Inference Modeling (Step 2)

Following the feature engineering step, we employed supervised learning to build predictive and inference models A and B. Results are presented in the following paragraphs.

**Figure 5:**
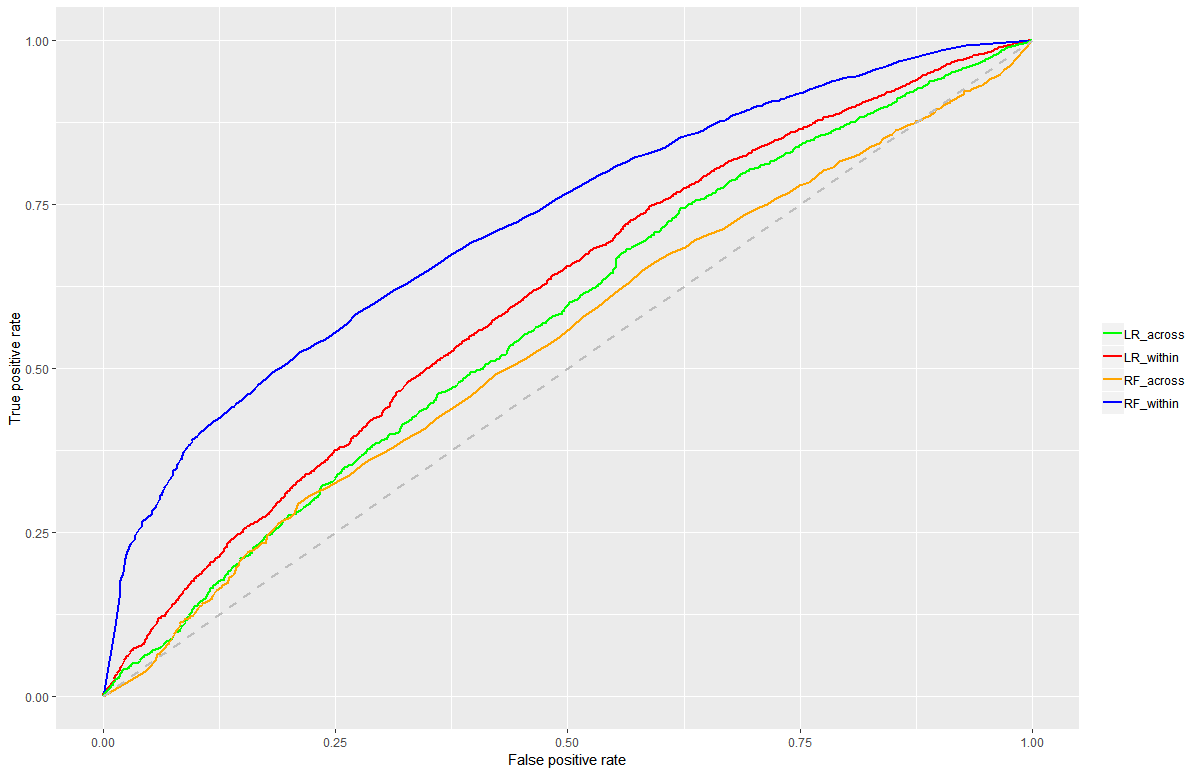
ROC curves for logistic regression (LR) and random forest (RF). The line color indicates whether the model was learned within- or across-users.

Model A—inferring symptoms over time: To demonstrate the capabilities of DHITA, we analyzed the predictive power of the various text features on the occurrence of a binge eating episode, a core ED behavior, within a 24-hour time window. For each intervention snippet, we determined the reported binge eating behavior closest in time, that is, either before or after the text. In this procedure, 37,228 snippets were matched with 5822 symptom severity reports. At this point of the analysis, various supervised learning methods such as neural networks or support vector machines could be used. As we do not aim to comparatively evaluate different methods, we chose logistic regression (LR) as a well-known method and random forest (RF) as a very powerful algorithm. For the RF training, we allowed for 200 individual trees, each with a maximum of 20 selected features. To support independent evaluation, we split the interventions snippets into training and test data, using 2 approaches. First, we randomly selected 70.00% (26,060/37,228) of all intervention snippets as training data, without accounting for the fact that they belong to different users. In doing so, we could expect that the training data and the test data contained intervention snippets for all users (we call this within-user learning). Second, we split the users into 2 groups; one was used for training, the other was used for testing purposes. This is called across-user learning, as we estimated the model on a separated set of users and could then apply it to new users. The receiver operating characteristic (ROC) curves are determined based on the test data (Figure 5). An area under the curve (AUC) of 0.72 for the within-user learning based on the RF algorithm demonstrates that the intervention snippets can be used to infer the binge eating episodes over time. For the across-user learning, the RF appeared to overfit, and the LR yielded better results (AUC=0.57). The ROC results can inform personalized microinterventions on the user level, for instance, identifying certain users prone to greater binge eating during the intervention based on their writing style and offering more individualized feedback (eg, a short online chat with the coach) or higher level of care. In summary, the results indicate that inferring symptom severity levels for known users (and unseen text snippets from these users) works significantly better than for users that have not been seen or, technically speaking, have not been included in the training data. As a result, models of type A might not be suited to inform early treatment decisions for incoming users.

Model B—predicting therapeutic outcome: To give an example for a type B model, we want to examine whether the baseline symptom level and the text features of the user-coach messages predict the symptom severity at the 6-month follow-up, as indicated by the Eating Disorder Examination Questionnaire global score [42]. As discussed above, we aggregated the text features on the user level, which led to 220 aggregated features per user and included (the numbers in parentheses indicate the number of features included):

- Metadata (5): total word count, total character count, number of messages, mean message length, and the number of messages per day
- Communication (2): average response rate and time
- Word usage (79): mean value for all terms
- Word embeddings (50): mean value for all dimensions
- POS (44): mean value for all word types
- Topic (10): mean value for topic features
- Sentiment (30): mean value and SD (this is included based on the hypothesis that variability in sentiments might have an influence on the therapeutic outcome) for all sentiment scores.

As demonstrated for the sentiment features, the list can easily be extended by applying other aggregation functions. Finally, we selected those users that had reported both their baseline and 6-month follow-up symptoms and had also sent more than 2 messages to their coaches. This resulted in 100 users.

**Figure 6:**
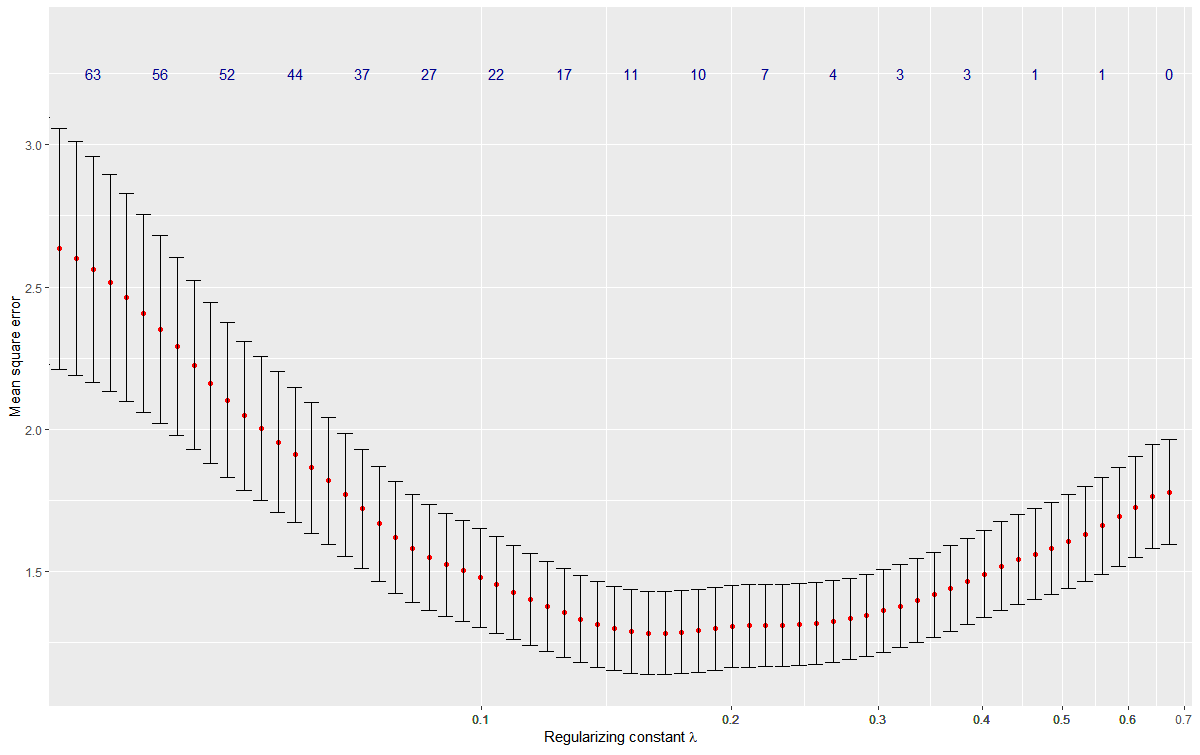
Cross-validation curve as a function of the regularizing constant λ. Error bars indicate the standard deviation for 100 folds in cross validation. The blue numbers indicate the number of non-zero parameters from the LASSO regression.

For the feature selection, we apply LASSO regression [43] with 50-fold cross-validation using the R package *glmnet* (Figure 6; for additional context, please refer to the article by Friedman et al [44] for a typical output plot of a LASSO regression). The analysis suggests that the mean square error (MSE) of the regression decreases while the regularizing constant λ increases. When the MSE reaches its minimum at λ∼0.15, 10 features are selected: the number of messages, the response rate, 4 specific words (*body, help, program,* and *let*), 3 POS tags (nouns, possessive endings, and pronouns that start with *wh*), and the baseline symptom level. When λ increases, additional features drop out until at 0.7 only the constant intercept term is left. At this point, the MSE is roughly 2 SDs above its minimum, indicating that the selected features have some predictive power. However, owing to the limited number of users included in this analysis, this pilot study was not adequately powered to identify text features that significantly predict outcome.

Note that in our case study, we do not make use of model type C, as this would require having additional characteristics associated with each text snippet, which we do not have.

## Discussion

### Principal Findings

Textual data can provide rich information that has the potential to expand the current insights of whether DHIs work, for whom, and in which circumstances. NLP, enhanced by machine learning techniques and statistical packages such as DHITA, may become a prominent tool to increase the intervention efficacy and to provide user-specific models to assist with clinical decision making. As dissemination efforts direct our field toward developing semiautomated and fully automated therapeutic platforms (eg, chatbots), text analysis is poised to inform such future initiatives. In this paper, we examined the use of text features to model and predict symptom severity over time for individual users.

DHITA offers an innovative approach to automating text analytics in DHIs. When we implemented this technical framework into the study of a DHI for EDs, preliminary results indicated that, using text features, DHITA was able to predict binge eating behaviors across and within users. The models developed in the test case of the HBI study are predictive as indicated by the AUC values; however, their clinical utilization is unclear. This approach could be further extended by integrating the quantitative diary entries (eg, number of meals and binge eating episodes) and the user information collected passively (eg, user location data and time of their activity in the program), which we have yet to incorporate into DHITA.

Some caveats to the model presented here should be mentioned. First, the predictive power of the 2 statistical models developed within the case study is weak. The models’ efficacy in predicting the intervention outcome is limited owing to the small number of users involved. A more rigorous test of the model in predicting outcome will require larger datasets. Second, we have described the type of features that are currently implemented in DHITA. This set can be extended in many ways (eg, readability, named entity recognition, and seeded topic models). Third, as this pilot study focused on text data exclusively, the models did not incorporate other empirically based markers of symptomatic change. Future studies should aim to identify how such variables interact with text data to help identify clinically useful predictors of engagement and outcome. Finally, we encourage future studies to test the proposed models in an experimental setting to inform therapeutic decisions.

### Conclusions

Text data enrich and expand our knowledge of the individuals presenting and utilizing psychological services provided digitally. The work reported here is innovative in several ways. First, we present DHITA, a technical framework to incorporate text data in analyzing and predicting key outcomes in large DHIs. Second, to the best of our knowledge, we demonstrate for the first time a method that applies word embeddings into the analysis of intervention outcomes. Third, we supplement the framework presented here with a case study, presenting data from a large RCT with numerous text snippets [40,41]. Fourth, by applying DHITA to this dataset, we were able to demonstrate that the text features predicted symptom changes over time.

Although the work presented in this paper is still preliminary, we encourage other teams to test the potential applicability of the framework in therapeutic decision making. Offering DHIs that are highly accessible, scalable, cost-effective, and evidence-supported, while integrating and empathetically responding to individual users’ unique preferences, characteristics, and history, will support global mental health care efforts and help reduce the burden of mental disorders.

## Abbreviations

AUC: area under the curve
DHI: digital health intervention
DHITA: Digital Health Interventions Text Analytics
ED: eating disorder
HBI: Healthy Body Image
LASSO: least absolute shrinkage and selection operator regression
LDA: Latent Dirichlet Allocation
LR: logistic regression
MSE: mean square error
NLP: natural language processing
POS: part-of-speech
RCT: randomized controlled trial
ROC: receiver operating characteristic
RF: random forest
SBED: Student Bodies–Eating Disorders

## References

[1] Carlbring P, Andersson G, Cuijpers P, Riper H, Hedman-Lagerlöf E (2018). Internet-based vs face-to-face cognitive behavior therapy for psychiatric and somatic disorders: an updated systematic review and meta-analysis. Cogn Behav Ther.

[2] Bakker D, Kazantzis N, Rickwood D, Rickard N (2016). Mental health smartphone apps: review and evidence-based recommendations for future developments. JMIR Ment Health.

[3] Price M, Yuen EK, Goetter EM, Herbert JD, Forman EM, Acierno R, Ruggiero KJ (2014). mHealth: a mechanism to deliver more accessible, more effective mental health care. Clin Psychol Psychother.

[4] Mohr DC, Burns MN, Schueller SM, Clarke G, Klinkman M (2013). Behavioral intervention technologies: evidence review and recommendations for future research in mental health. Gen Hosp Psychiatry.

[5] Spiranovic C, Matthews A, Scanlan J, Kirkby KC (2016). Increasing knowledge of mental illness through secondary research of electronic health records: opportunities and challenges. Adv Mental Health.

[6] Vorderstrasse A, Lewinski A, Melkus GD, Johnson C (2016). Social support for diabetes self-management via eHealth interventions. Curr Diab Rep.

[7] (2014). Clinical Trials.

[8] Cook BL, Progovac AM, Chen P, Mullin B, Hou S, Baca-Garcia E (2016). Novel use of natural language processing (NLP) to predict suicidal ideation and psychiatric symptoms in a text-based mental health intervention in Madrid. Comput Math Methods Med.

[9] Dinkel H, Wu M, Yu K (2019). arXiv e-Print archive.

[10] Hirschberg J, Manning CD (2015). Advances in natural language processing. Science.

[11] Baek H, Ahn J, Choi Y (2012). Helpfulness of online consumer reviews: readers' objectives and review cues. Int J Electron Comm.

[12] Hoogendoorn M, Berger T, Schulz A, Stolz T, Szolovits P (2017). Predicting social anxiety treatment outcome based on therapeutic email conversations. IEEE J Biomed Health Inform.

[13] Tran T, Luo W, Phung D, Harvey R, Berk M, Kennedy RL, Venkatesh S (2014). Risk stratification using data from electronic medical records better predicts suicide risks than clinician assessments. BMC Psychiatry.

[14] Nobles AL, Glenn JJ, Kowsari K, Teachman BA, Barnes LE (2018). Identification of Imminent Suicide Risk Among Young Adults using Text Messages. Proc SIGCHI Conf Hum Factor Comput Syst.

[15] Ranard BL, Werner RM, Antanavicius T, Schwartz HA, Smith RJ, Meisel ZF, Asch DA, Ungar LH, Merchant RM (2016). Yelp reviews of hospital care can supplement and inform traditional surveys of the patient experience of care. Health Aff (Millwood).

[16] Pestian J, Nasrallah H, Matykiewicz P, Bennett A, Leenaars A (2010). Suicide note classification using natural language processing: a content analysis. Biomed Inform Insights.

[17] Haerian K, Salmasian H, Friedman C (2012). Methods for identifying suicide or suicidal ideation in EHRs. AMIA Annu Symp Proc.

[18] Coppersmith G, Dredze M, Harman C (2014). Quantifying Mental Health Signals in Twitter. Proceedings of the Workshop on Computational Linguistics and Clinical Psychology: From Linguistic Signal to Clinical Reality.

[19] Althoff T, Clark K, Leskovec J (2016). Large-scale analysis of counseling conversations: An application of natural language processing to mental health. Transact Assoc Comput Linguist.

[20] Miner AS, Milstein A, Schueller S, Hegde R, Mangurian C, Linos E (2016). Smartphone-based conversational agents and responses to questions about mental health, interpersonal violence, and physical health. JAMA Intern Med.

[21] Fox S, Duggan M (2013). Pew research center.

[22] Fitzpatrick KK, Darcy A, Vierhile M (2017). Delivering cognitive behavior therapy to young adults with symptoms of depression and anxiety using a fully automated conversational agent (Woebot): a randomized controlled trial. JMIR Ment Health.

[23] Bremer V, Becker D, Funk B, Lehr D (2017). Predicting the individual mood level based on diary data. Proceedings of the 25th European Conference on Information Systems.

[24] Kohlschütter C, Frankhauser P, Nejdl W (2010). Boilerplate Detection Using Shallow Text Features. Proceedings of the third ACM international conference on Web search and data mining.

[25] Manning CD, Raghavan P, Schütze H (2008). Introduction To Information Retrieval.

[26] Zhong N, Li Y, Wu ST (2012). Effective pattern discovery for text mining. IEEE Trans Knowl Data Eng.

[27] Bengio Y, Ducharme R, Vincent P, Jauvin C (2003). A neural probabilistic language model. J Mach Learn Res.

[28] Mikolov T, Sutskever I, Chen K, Corrado GS, Dean J (2013). Distributed Representations of Words and Phrases and Their Compositionality. Proceedings of the 26th International Conference on Neural Information Processing Systems - Volume 2.

[29] Pennington J, Socher R, Manning C (2014). Glove: Global Vectors for Word Representation. Proceedings of the 2014 Conference on Empirical Methods in Natural Language Processing.

[30] de Boom C, van Canneyt S, Demeester T, Dhoedt B (2016). Representation learning for very short texts using weighted word embedding aggregation. Pattern Recogn Letters.

[31] Martinez AR (2012). Part-of-speech tagging. WIREs Comp Stat.

[32] Marcus PM, Marcinkiewicz MA, Santorini B (1993). Building a large annotated corpus of English: the Penn Treebank. Comput Linguist.

[33] Nadeau D, Sekine S (2007). A survey of named entity recognition and classification. Lingvist Investigat.

[34] Blei DM (2012). Probabilistic topic models. Commun ACM.

[35] Mohammad SM, Turney PD (2010). Emotions Evoked by Common Words and Phrases: Using Mechanical Turk to Create an Emotion Lexicon. Proceedings of the NAACL HLT 2010 Workshop on Computational Approaches to Analysis and Generation of Emotion in Text.

[36] Pitler E, Nenkova A (2008). Revisiting Readability: A Unified Framework for Predicting Text Quality. Proceedings of the 2008 Conference on Empirical Methods in Natural Language Processing.

[37] Michalke M (2017). The Comprehensive R Archive Network.

[38] Ohms J (2017). The Comprehensive R Archive Network.

[39] Becker D, van Breda W, Funk B, Hoogendoorn M, Ruwaard J, Riper H (2018). Predictive modeling in e-mental health: a common language framework. Internet Interv.

[40] Fitzsimmons-Craft EE, Balantekin KN, Eichen DM, Graham AK, Monterubio GE, Sadeh-Sharvit S, Goel NJ, Flatt RE, Saffran K, Karam AM, Firebaugh M, Trockel M, Taylor CB, Wilfley DE (2019). Screening and offering online programs for eating disorders: reach, pathology, and differences across eating disorder status groups at 28 U.S. universities. Int J Eat Disord.

[41] Fitzsimmons-Craft E, Firebaugh ML, Graham AK, Eichen DM, Monterubio GE, Balantekin KM, Karam AM, Seal A, Funk B, Taylor CB, Wilfley DE (2019). State-wide university implementation of an online platform for eating disorders screening and intervention. Psychol Serv.

[42] Fairburn CG, Beglin SJ (1994). Assessment of eating disorders: interview or self-report questionnaire?. Int J Eat Disord.

[43] Hastie T, Tibshirani R, Friedman J (2001). The Elements of Statistical Learning.

[44] Friedman J, Hastie T, Tibshirani R (2010). Regularization paths for generalized linear models via coordinate descent. J Stat Softw.

